# Subjective age, worry and risk-related perceptions in older adults in times of a pandemic

**DOI:** 10.1371/journal.pone.0274293

**Published:** 2022-09-29

**Authors:** Maiken Tingvold, Isabelle Albert, Martine Hoffmann, Elke Murdock, Josepha Nell, Anna E. Kornadt

**Affiliations:** 1 Department of Behavioral and Cognitive Sciences, University of Luxembourg, Esch-sur-Alzette, Luxembourg; 2 GERO Competence Center for Ageing, Itzig, Luxembourg; ISEG Lisbon School of Economics and Management, PORTUGAL

## Abstract

During the Covid-19 pandemic, older people have been in the spotlight of the public debate. Given their higher risk of severe outcomes of the disease, they have been described as especially vulnerable and as a burden to others and society. We thus wanted to investigate how older people’s perception of their own age, that is their subjective age, as well as their Covid-19 related risks and worries were related during the pandemic and whether these relationships varied according to participants’ subjective health. We used data from the longitudinal CRISIS study which was conducted in the Grand-Duchy of Luxembourg in June and October 2020. Participants were aged 60–98 and responded on questionnaires regarding their subjective age, worry of falling ill with Covid-19, perceived risk of contracting the virus, perceived risk of falling seriously ill if they contracted Covid-19, as well as their subjective health and covariates. Three cross-lagged panel models were constructed to explore the longitudinal, bidirectional relationships between the variables. Cross-sectionally, a higher subjective age was related to more perceived risk of a serious course of disease. Longitudinally, subjective age and worry did not show any significant association over time, and neither did subjective age and perceived risk of contracting the virus. However, subjective health significantly moderated the relationship of worry and subjective age, showing different trajectories in the relationship depending on whether subjective health was good or bad. Higher perceived risk of falling seriously ill increased subjective age over time. Again, subjective health moderated this relationship: the perceived risk of falling seriously ill affected subjective age only for those with better subjective health. Our findings show the interactive relationship between subjective age and Covid-19 related cognitions and emotions and provide guidance for identifying older people that are most susceptible for negative age-related communication during the pandemic.

## Introduction

The current pandemic spreading through our societies has put a spotlight on the older generations. The main reason for this increased attention is that on average the risk of becoming seriously ill or dying from Covid-19 increases with chronological age [1] and many public measures have been taken to shield older people from exposure to the virus. Due to the associated risk of a severe course of the disease and mortality that comes with higher age, older people have been portrayed as highly vulnerable and in need of protection and care [2], sometimes irrespective of their own wishes. Related is also the narrative that older people are seen as a burden to society due to the high amount of medical resources they require in already stretched health care systems, in addition to the effort that other population groups need to invest in protecting this vulnerable group [2]. These narratives of vulnerability and burden neglect the fact that older people represent a highly heterogeneous group: increased risks neither apply directly to each older person as we observe large individual differences in health status in later life, nor are older people universally vulnerable but can contribute to societal efforts in combatting the Covid-19 crisis. Experts on aging have raised questions regarding secondary impacts on older people of both the public debate and the safety measures, such as an increase in ageism and the related effect on older people’s wellbeing [2–5]. Therefore, we need empirical evidence on how this discourse regarding aging affects older adults themselves and how their own experience of age as well as their own experience of risks and worries interact. Given the prominent focus on older people in the pandemic discourse, we aim to investigate the reciprocal relationship between the age people feel, i.e., their subjective age, their worries as well as their perception of their own risks regarding Covid-19 during this historical time.

### Subjective age and the pandemic discourse

Although chronological age has been closely linked to many health outcomes, it does not reflect the aging process in its entirety [6], as subjective experiences of aging are highly important for development [7]. Subjective age, for instance, refers to how old people feel in relation to their chronological age [8]. It reflects how people view their own aging process and is thus a part of their age identity [9]. From a developmental perspective, subjective age shows a distinct lifespan trajectory: in earlier life people feel generally older than their actual age and as they grow older they feel increasingly younger [8,10]. Subjective age can be an indicator of different aging processes as it has been termed a “biopsychosocial marker” of aging [11]. It has shown to be sensitive to both biological and psychosocial changes, such as stressor exposure and biological aging, which not only increase the age people feel on a daily basis but also over time [12,13].

In terms of predictive relevance for development, a lower subjective age is also associated with beneficial outcomes for middle-aged and older adults: feeling younger than one’s chronological age, at least to a certain degree [14], is longitudinally related to physical health factors such as better physical health, health behaviour and self-rated health [15–17]. A meta-analysis by Alonso Debreczeni and Bailey [9] found that a younger subjective age is associated with reduced depressive symptoms as well as increased subjective wellbeing and better cognitive performance. In addition, a recent study showed an effect of subjective age on the relations between psychological symptoms; people with an older subjective age had a stronger effect of anxiety sensitivity on anxiety and depression [18].This influence of subjective age on outcome variables has also been corroborated by experimental studies. For instance, after receiving positive feedback from a hand-grip strength test, participants reported a younger subjective age and increased their performance on a second test, compared to participants who received no feedback [16]. In sum, this shows that subjective age can serve as both predictor and outcome in relation to variables like health and stress and, that overall, a younger subjective age is linked to better health and wellbeing in later life.

In times of a pandemic, in which age plays a major role in terms of actual and perceived risk, risk communication, media discourse and policy decision making, subjective age remains highly relevant and important to study. On the one hand, subjective age can serve as a coping mechanism for older people, allowing to distance oneself from ones’ actual age and the related risks and worries, especially when belonging to the group of older people has negative implications [19]. This distancing effect would mean that people who feel threatened by being placed into the risk group of “older people” could cope with this challenge by distancing themselves from this group (e.g., by social comparisons, or stressing their “younger” characteristics), which might ultimately result in a lower subjective age. Previous studies have indeed shown that subjective age had a protective function when facing aging-related stereotypes, protecting the individual from negative views and vulnerabilities [20,21], or when feeling lonely due to the pandemic and psychiatric symptoms [22]. Another study showed that when Covid-19 infection rates went up peoples’ subjective ages decreased [23], suggesting a distancing effect when risk is especially high. Interestingly, the effect was stronger for older individuals and for people who reported stronger health concerns. Finally, older people with more positive self-perceptions of aging appeared more resilient during the pandemic outbreak, indicating that subjective experiences of own aging can play an important role in protecting the individual from negative outcomes caused by the pandemic [24].

However, there is also reason to assume that exposure to the age-related issues in the current pandemic might lead to people feeling older. In daily diary studies, individuals reported older subjective ages on days with more stress- and negative affective experiences [25] and perceived stress seems to increase subjective age over longer periods of time [26]. Such experiences may be more frequent in the pandemic situation. Earlier studies have also shown associations between prolonged stress exposure and traumatic experiences with accelerated subjective aging [27]. These findings give rise to the idea that subjective age might increase in the current pandemic situation. Furthermore, the pandemic discourse could also make age and one’s belonging to a certain risk group more salient (see also [28]), thereby making older people face their own vulnerability, ultimately resulting in older subjective ages.

These competing hypotheses, the distancing- and the stress hypothesis, were first brought forward and tested in a study by Terraciano and Stephan [29] who at the beginning of 2020 (January to April), investigated the trajectories of subjective age in a large US sample of persons aged 18 years or older. The authors also investigated predictors of change in subjective age and found that people felt younger over the course of this early stage in the pandemic. The change in subjective age was predicted by the belief that the Corona virus is especially threatening to older people. In other words, the results align with the distancing hypothesis as a stronger perception of threat was predicting a younger subjective age. However, this study did not follow-up on participants after the initial phase of the pandemic, and it did not explicitly link subjective age with personal assessments of risk and worry. In addition, the study included both younger and older adults and did not focus especially on those who are most likely affected by the negative pandemic discourse. Besides, the study did not investigate bidirectional relationships, acknowledging that a younger subjective age might also be a resource which might ameliorate fears and age-related threatening situations [19]. Furthermore, ones’ health status most likely plays a role for subjective age during the pandemic and the relation to risk (perceptions) and worries.

### Subjective age, worry and risk perception

Given the increased risk of severe progression of disease and mortality from Covid-19 in higher chronological ages [30], which was also highly present in the pandemic discourse, we wanted to investigate the link of subjective age to worry about Covid and to the perceived risk of contracting the virus and the risks of becoming seriously ill.

In general, worry is described as a negative chain of cognitions that represents an attempt at mental problem solving of an issue, whose outcome is unknown and (thus) has the possibility of being negative [31]. According to worry is stipulated by the anticipation of negative and uncertain outcomes [32], and in such, a state of emotions related to how one perceives the situation. Maxfield and Pituch [33] found that worry about Covid-19 does indeed increase with chronological age and in a study by Inbar and Shinan-Altman [34], a negative association between age and emotional reactions to the pandemic was found. This is in line with other findings indicating that high-risk populations show more severe psychological reactions to the pandemic [34]. Subjective age might be a central factor in this relationship, since feeling older might manifest the perception of being part of the risk group and thereby, increase personal relevance and thus heightened worries. Indeed, a recent study by Greenblatt-Kimron, Ring [35] showed that Covid-19 related worries predicted peritraumatic stress, with an effect stronger for those who felt they were getting older faster. Another recent study by Ayalon and Cohn-Schwatz [36] showed that self-perceptions of aging and perceived age-based discrimination in healthcare settings were significant predictors of health worries related to Covid-19.

Closely related to one’s worries are the risk perceptions one has of a situation. People have in general been concerned about Covid-19 [37,38], yet they underestimate their own risk. A study based on a US adult population showed that people underestimate their absolute and relative fatality risk compared to epidemiological figures [39]. Given that higher age is indeed linked to an increased likelihood of falling seriously ill with Covid-19, how likely people think it could be that they would contract the virus and how sick they think they would become, should also depend on their perceived belonging to the risk group. In a study by Bruine de Bruin [40], older chronological age was indeed associated with greater perceived risk of dying if getting ill with the Corona virus, but the perceived risk was considered lower for contracting the virus. Given that subjective age might be an even better indicator of one’s perception of belonging to a risk group, our results could be in line with those of Bruine de Bruin [40], where older subjective ages are related to greater risk of severe Covid-19 illness but less with the risk perception of contracting the virus.

### The role of bidirectional relationships

Given the importance of subjective age as a predictor of wellbeing and health, factors affecting subjective age have also been of interest in previous studies. Psychological, social and biomedical factors like perceived age discrimination and reduced grip strength can explain variations in subjective age [12]. As noted above, the relationship between subjective age, worry and risk perceptions is open to three possibilities: a) subjective age as an antecedent of worry and risk perceptions, as feeling older might make people more susceptible for age-related negative cognitions and emotions; b) as subjective age can be a marker of developmental processes, and increased worry and risk perception might increase negative affect and be indicative of an older identity, worry and risk perceptions could be antecedents for subjective age, c) given that both processes can co-exist, a bidirectional relationship, in which subjective age predicts worry and risk perception and vice versa is also plausible.

Empirically, only a few studies have explored the bidirectionality of subjective age and its relation to other constructs so far. Spuling, Miche [17] investigated the relationship between different subjective age dimensions and health status measures. A more recent study explored the bidirectional relationship between subjective age and depressive symptoms and activities of daily living [41]. Both studies concluded that subjective age was the antecedent of the other constructs. Wettstein and colleagues [26] on the other hand, found a bidirectional relationship in their study linking subjective age and perceived stress. Thus, more studies are needed in order fully understand the bidirectional relationships of subjective age and other variables.

### The moderating role of health status

Compromised physical health is a major factor for severe outcomes of a Covid-19 infection [42,43], and underlying health conditions are also partially responsible for the higher risk of older adults for severe courses of the disease [44]. Therefore, health needs to be considered when investigating worry and risk perceptions regarding Covid-19, especially in older people. An indicator for health processes is perceived, self-rated or subjective health, which has high predictive value for mortality [45], and has also been linked to subjective age [46]. In the pandemic context, Inbar and Shinan-Altman [34] found that emotional reactions to the pandemic were higher for those who rated their health status as lower. In line with these results there is reason to believe that ones’ health status could also moderate the relationship of worry and subjective age: Those with higher subjective age and worse health status might feel especially worried and at risk, because they combine two risk factors (belonging to an older age group and worse health status) and those who perceive their health status as worse might have more problems distancing themselves from the risk group when perceiving threat, since worse perceived health might be a factor that is associated with belonging to an older age group.

### Aim and hypotheses

Given the importance of age in the actual risk and the risk communication to the public during the Covid-19-pandemic, as well as the importance of subjective age for health and well-being in later life, the current study sets out to investigate the associations between worry, perceived risks, health status and subjective age among older people during the first year of the pandemic [33,40,43]. To the best of our knowledge, previous studies have not linked subjective age directly to risk perceptions and worries over time in a bidirectional way and have not addressed the moderating role of ones’ health status.

Our first hypothesis is that worry and perceived risks (contracting the virus, falling seriously ill) will affect how old people feel. In line with the findings by Terraciano and Stephan [29] as well as Wahl and Wettstein [23], we assume that older people will distance themselves from their actual age over time in the context of the Covid-19 pandemic, if they have more Covid-related worry and a higher risk perception: Those who feel more worried or perceive higher risk of infection or falling seriously ill at first measurement, will feel younger three months later, to psychologically remove themselves from the risk group (cf. [19,21]).

Our second hypothesis is targeting how subjective age affects how worried older people are and how much risk they perceive. Given that people might base their worries and risk perceptions on the age they feel like, and that distancing from the older age group might be a protective mechanism, we expected that people who feel older at the first measurement point should worry more three months later and perceive more risk of infection and of falling seriously ill.

Thirdly, we expect subjective health to moderate the relationships. Given that a lower health status might hamper distancing from the at-risk group, we expect that the distancing effect (i.e., feeling younger) due to heightened worry and risk perception will only emerge if people are in good health. For the other direction, we assume that those who feel younger will feel even less worried and at risk if they perceive themselves to be in good health. Here we also expect a particularly strong moderation effect for the relationship between subjective age and perceived risk of falling seriously ill, as the latter should be more closely related to ones’ health status.

## Methods

### Sample and procedure

We use data from the CRISIS study which was conducted in the Grand-Duchy of Luxembourg in June and October 2020, which represented a time between the first and second pandemic wave, when some relaxations of sanitary measures had already been in place. At the first wave a total of *N =* 611 community-dwelling, older participants from Luxemburg were recruited by a survey research institute (TNS ILRES). Participants were aged 60–98 (Mean = 69.92, SD = 6.97), and 49.6% were female. The survey was conducted partly by phone through random digit dialling (*n* = 240, response rate 27%), and partly online, recruiting people from a database of Luxembourgish residents who had agreed to be contacted for online surveys (*n* = 371, response rate 40%). The participants responded to questions on socio-demographic information, their concerns about the Covid-19 crisis in Luxembourg in general, their personal situation during the crisis, subjective age, worries and a variety of other risk and resilience factors. Informed consent was given verbally for those who participated by phone, and in written format for those participating online. The study was approved by the Ethics Review Panel of the University of Luxemburg (ERP 20-042-C CRISIS). Participants were invited for a second wave in October, in which *N* = 523 persons participated. For more details on the sample, procedure, and assessment, see Kornadt et al. [28].

### Measures

#### Subjective age

Subjective Age (SA) was assessed at both timepoints (SA T1 and SA T2, respectively) by asking people “Aside from your actual age: how old do you feel in years?”. Participants’ chronological ages were then subtracted from participants’ felt age, so that negative values indicate feeling younger. Following recommended practice, outliers, i.e., values that were three standard deviations above or below the mean were removed (T1: more than 38 years younger or more that 18 years older, 1.3% of cases; T2: more than 37 years younger or more than 19 years older, 0.8% of cases).

#### Subjective health

Subjective health (SH) was measured at the first timepoint by asking, “How would you rate your current state of health?”, and participants indicated their subjective health on a five-point scale ranging from “very good” to “very bad”.

#### Worry about falling ill with Covid-19

Worry was measured at both timepoints (WORRY T1 and WORRY T2) by asking participants to indicate how much they agreed with the statement “I am worried about falling ill with Covid-19”, giving their response on a four-point scale ranging from “totally agree” to “absolutely not agree”.

#### Perceived risk

Perceived risk at both timepoints was assessed by two questions, related to the risk of falling ill (PRISK T1 and PRISK T2), and to the risk of developing a serious course of disease (PRISK-S T1 and PRISK-S T2). The former was measured by asking participants “To which extent do you estimate the risk of yourself falling ill with Covid-19?” and to indicate their responses on a five-point scale ranging from “very likely” to “very unlikely”. PRISK-S was assessed with the question “If you would fall ill with Covid-19, how likely do you think it is that you would develop a serious course of disease?”. Participants again gave their responses on a five-point scale ranging from “very likely” to “very unlikely”. For both sets of questions, participants could indicate whether they had already contracted the virus and those who answered in the affirmative (T1: *N* = 8, T2: *N* = 5) were excluded from further analyses.

Finally, participants also reported their chronological age, gender (1 = male, 2 = female), and level of education (higher values represents higher qualifications).

### Analyses

**Fig 1:**
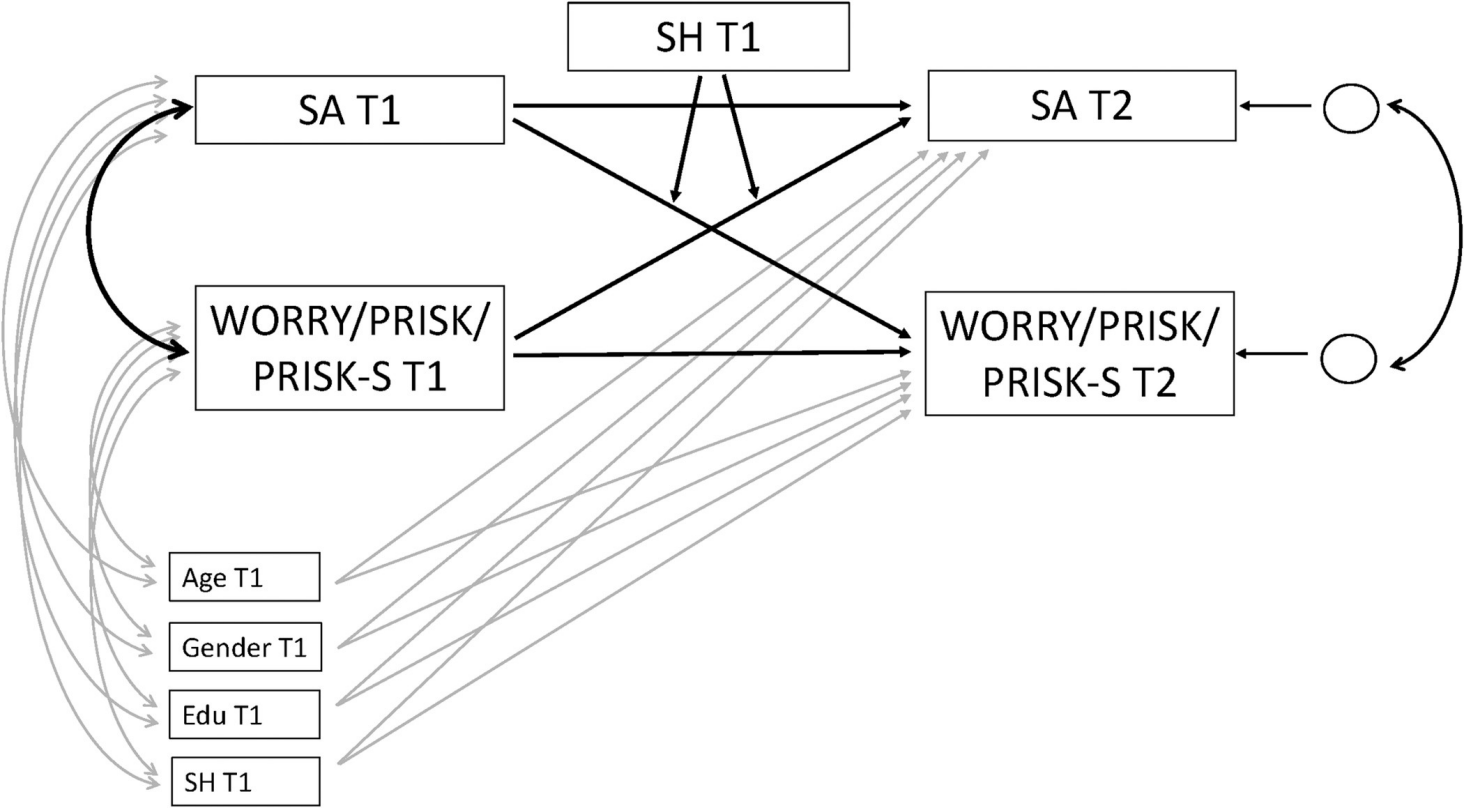
Cross-lagged panel model showing the bidirectional, longitudinal relationship between subjective age (SA) and the three variables worry (WRY), risk of falling ill (PRISK), and risk of serious course of disease (PRISKS), including all covariates (bivariate correlations between covariates are not displayed for reasons of parsimony). Please note that separate models were run for all three variables. T1 = timepoint 1; T2 = timepoint 2; Edu = Education.

Descriptive statistics and bivariate correlations were computed with SPSS 26 to address means and bivariate relationships between variables. All variables were recoded so that higher values indicate higher endorsement. To examine the longitudinal, bidirectional relationships between subjective age, worry and risk perceptions we computed separate cross-lagged panel models for each variable (Fig 1) using Mplus 8 [47]. We first estimated the model including only subjective age and the respective worry/risk variables. In a second step, chronological age, education, and gender were added as covariates. We allowed correlations of the covariates with the T1 predictors and used the covariates as predictors of the T2 outcome variables (Fig 1). In a third step, subjective health was introduced as a moderator for the longitudinal paths. Before computing interaction terms, all variables were standardized.

## Results

### Descriptive statistics and bivariate correlations

Descriptive statistics and bivariate correlations for all variables are presented in Table 1. On average, people felt younger than their chronological age. Mean values of subjective age increased over time (*t* (448) = -4.302, *p <* .001), indicating that on average, people felt somewhat older at the second compared to the first timepoint. Subjective age was significantly and positively related to perceived risk of serious disease at both timepoints indicating that people who felt older also perceived higher risk of serious disease. There was also a significant and negative relation between subjective age and subjective health, those who felt older also perceived their health to be worse. However, there was no significant correlation between subjective age and worry. Worry at timepoint 1 had a small but significant negative correlation with perceived risk of getting infected at timepoint 1, indicating that those who worried more perceived their risk as lower. Subjective health was negatively correlated with risk of serious disease at both timepoints, but only to risk of contracting the virus at timepoint 1, implying that those experiencing worse health perceived their risks as higher.

**Table 1:** Descriptive statistics and bivariate correlations for all study variables at both time points.

Variable	*N*	*M*	SD	1	2	3	4	5	6	7	8	9	10	11
1. SA T1	532	-10.03	7.59	-										
2. Worry T1	601	2.396	0.84	0.02	-									
3. PRisk T1	566	2.224	0.80	-0.01	-.11*	-								
4. Prisk-S T1	540	-2.224	0.88	0.13*	-0.02	0.23*	-							
5. SH	609	1.98	0.72	-0.26*	0.01	-0.07*	-0.37*	-						
6. SA T2	503	-8.532	7.26	0.60*	-0.02	0.02	0.19*	-0.21*	-					
7. Worry T2	513	2.452	0.83	0.00	0.05	-0.09*	-0.08	0.06	0.05	-				
8. PRisk T2	489	2.269	0.80	0.00	0.00	0.46*	0.27*	-0.01	0.12*	-0.01	-			
9. Prisk-S T2	458	2.633	0.88	0.12*	0.11	0.17*	0.57*	-0.30*	0.17*	0.04	0.25*	-		
10. Age	608	69.92	6.97	-0.10*	0.04	-0.05	0.15*	-0.13*	-0.07	-0.05	-0.02	0.12*	-	
11. Edu	544	3.38	1.15	0.05	-0.01	-0.02	-0.07*	0.17*	0.10*	0.06	0.03	-0.09	-0.13*	-
12. Gender	611			0.01	-0.05	0.05	-0.05	0.01	-0.06	-0.03	-0.01	-0.04	-0.06	-0.16*

T1, Timepoint1; T2, Timepoint 2; Gender 1. Male; 2, Female; SA, Subjective Age; Prisk, Perceived Risk of contracting virus; Prisk-S, Perceived Risk of Serious. disease course; EDU, education.

* P = < .0.05.

### Longitudinal relation of subjective age and worry

**Fig 2:**
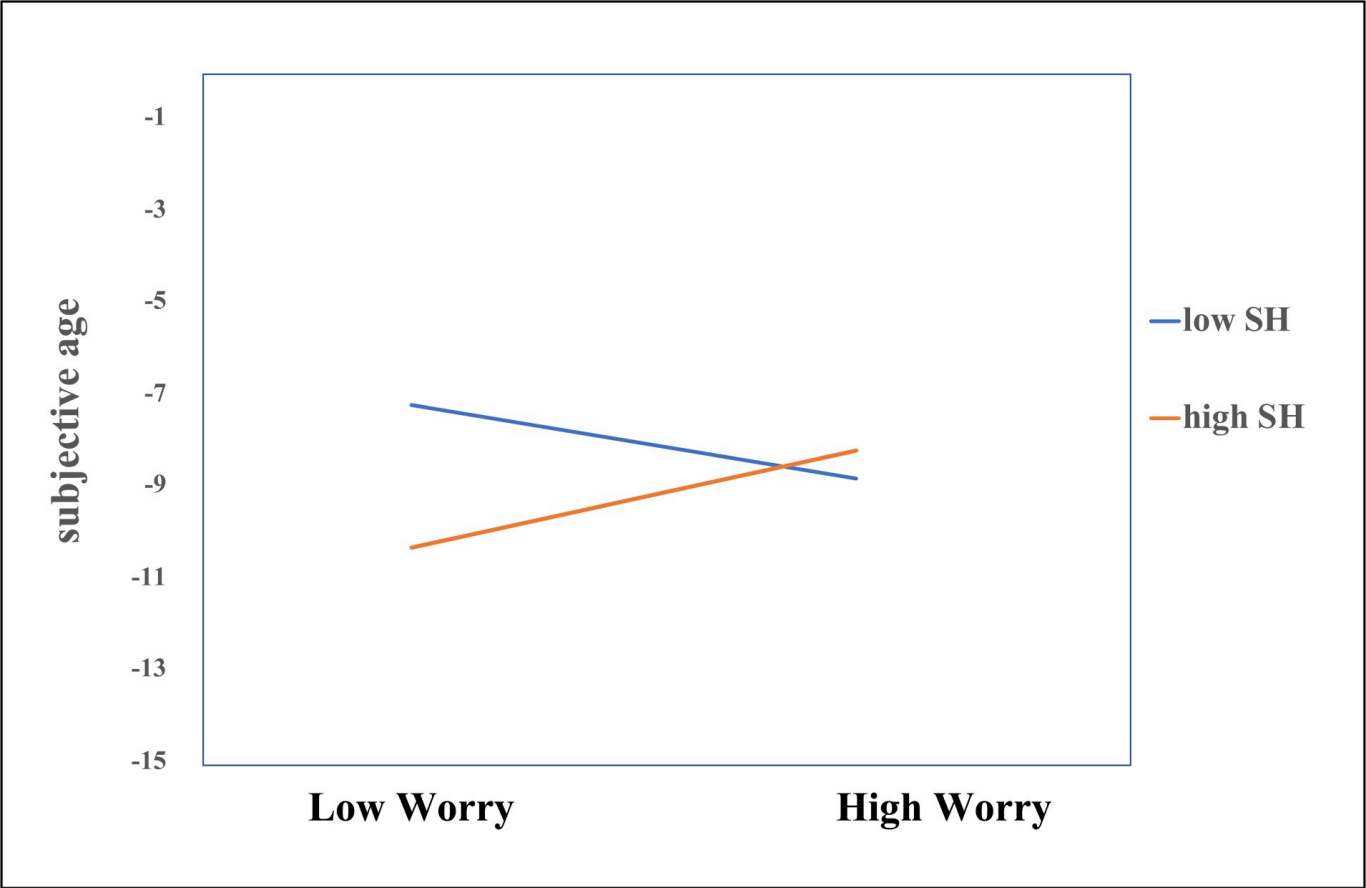
Simple slopes for the effect of worry at T1 predicting subjective age at T2, moderated by subjective health (SH). High and low worry and subjective health groups represent values 1 SD above and below the mean, respectively. Analyses are controlled for age, gender, education, and subjective health.

Model fit indices for all cross-lagged models are presented in Table 2. The first model with worry and subjective age showed a significant positive result for the longitudinal path from subjective age to worry (Table 3, Model 1), indicating that those with higher subjective age at T1 worried more at the second timepoint, but this significant effect disappeared when the covariates were entered into the model (Table 3, Model 2). The covariates age, education and gender themselves did not significantly predict any of the dependent variables (see Table 1 in S1 Appendix). When including subjective health as a moderator, subjective health positively moderated the longitudinal path from worry to subjective age (Table 3, Model 3), even in the presence of the covariates. Simple slope analyses showed that the path from worry to subjective age was significant for high and low health status (+/- SD above and below the mean, respectively), but differed in direction (Fig 2). For those with worse health status, a lower amount of worry at T1 predicted an older subjective age at T2 (*b* = 1.061, *p =* .*008)*. For those with a better health status, more worry at T1 predicted an older subjective age at T2 (*b* = -0.795, *p =* .*031)*. As shown in Fig 2, the difference between groups of high and low health groups was largest at lower levels of worry, while the effect was similar at high levels of worry. There was no significant correlation between the residuals of worry and subjective age at T2 in any of the models including worry and subjective age (simple model: *rc =* .05, *p* = .278), indicating that no correlated changes could be observed beyond the changes explained by the model.

**Table 2:** Model fit indices for the cross-lagged regression models including subjective age as well as worry (A), perceived risk of infection (B), and perceived risk of serious disease (C) across two time points.

	**X2 (df)**	***p***	**RMSEA (90%ClI)**	**CFI**	**SRMR**
Model 1: Simple model SA and Worry	369.668 (5)	0.00	0[0.00,0.00]	1.00	0.00
Model 1: Simple model SA and Worry, with covariates	386.424 (13)	0.00	0[0.00,0.00]	1.00	0.00
Model 1: Model with SH as moderator, with covariates	403.373 (17)	0.00	0.06 [0.01,0.11]	0.99	0.01
Model 2: Simple model SA and PRISK	326.046 (5)	0.00	0[0.00,0.00]	0.01	0.00
Model 2: Simple model SA and PRISK, with covariates	342.116 (13)	0.00	0[0.00,0.00]	0.01	0.00
Model 2: Model with SH as Moderator, with covariates	353.411 (17)	0.00	.075 [0.03,0.13]	0.98	0.16
Model 3: Simple model SA and PRISK-S	389.144 (5)	0.00	0 [0.00,0.00]	0.01	0.00
Model 3: Simple model SA and PRISK-S, with covariates	413.897 (13)	0.00	0 [0.00,0.00]	0.01	0.00
Model 3: Model with SH as moderator, with covariates	428.190 (17)	0.00	0.05 [0.00,0.01]	0.99	0.01

Model 1, subjective age and worry; Model 2, subjective age and perceived risk of infection; Model 3, subjective age and perceived risk of serious disease. RMSEA, root-mean-square-error of approximation; CFI, comparative fit index; SRMR, standardized root mean square residual. SA, Subjective Age; Prisk, Perceived Risk of contracting virus; Prisk-S, Perceived Risk of Serious course of disease.

**Table 3:** Standardized estimates for the cross-lagged regression models including subjective age and worry of falling ill with Covid-19.

	Initial Correlation	Stability		Crossed-lagged effect		Moderator effect		Direct effect		Residual Correlation	
CL Model with SA and Worry	ʳWorry1↔SA1	SA1↔SA2	Worry1→Worry2	Worry1→SA2	SA1→Worry2	Worry1→SA2	SA1→Worry2	SH→Worry2	SH→SA2	Worry2↔SA2	
1 Simple Model	.102*	.623*	.518*	0.021	.079*					.050	
2 Simple Model with Covariates	.103*	.605*	.511*	0.009	0.058					.039	
3 Model with Subjective Health	.111	.618*	.512*	0.018	0.071	0.137*	0.034	-0.058	-0.086*	.030	

Models with covariates include age, gender, education and subjective health at timepoint 1. Worry1, worry of falling ill with Covid-19 at timepoint 1; SA1, subjective age at timepoint 1; Worry2, worry of falling ill with Covid-19 at timepoint 2; SA2, subjective age at timepoint 2; SH, subjective health.

*p<0.*05*.

### Subjective age and perceived risk of Covid-19 infection

None of the cross-lagged models which contained subjective age and perceived risk of a Covid-19 infection yielded any significant longitudinal relationship, and there was also no moderation effect of subjective health (Table 4). The covariates age, education and gender did not significantly predict any of the dependent variables in any of the models including subjective age and perceived risk of infection (see Table 2 in S2 Appendix). There was a significant correlation between the residuals of the T2 variables in all models including subjective age and perceived risk of infection (model 1: *rc =* .15, *p* = .001), including the final model (Table 3, model 3), representing correlated changes irrespective of directional relationships: as subjective age increased, so did perceived risk of infection.

**Table 4:** Standardized estimates for the cross-lagged regression models including subjective age and perceived risk of infection.

	Initial Correlation	Stability		Crossed-lagged effect		Moderator effect		Direct effect		Residual Correlation	
CL Model, SA and Prisk	ʳPrisk1↔SA1	SA1↔SA2	Prisk1→Prisk2	Prisk1→SA2	SA1→Prisk2	Prisk1→SA2	SA1→Prisk2	SH→Prisk2	SH→SA2	Prisk2↔SA2	
1 Simple Model	-0.016	.624*	.489*	0.009	-0.013					.154*	
2 Simple Model with Covariates	-0.013	.605*	.489*	0.002	-0.005					.158*	
3 Model with Subjective Health	-0.012	.604*	.489*	-0.005	0	0.062	0.021	0.013	-0.079	.154*	

Models with covariates include age, gender, education and subjective health at timepoint 1. Prisk1, perceived risk of contracting the Corona virus at timepoint 1; SA1, subjective age at timepoint 1; Prisk2, perceived risk of contracting the Corona virus at timepoint 2; SA2, subjective age at timepoint 2; SH, subjective health.

*p<0.*05*.

### Subjective age and perceived risk of falling seriously ill with Covid-19

**Fig 3:**
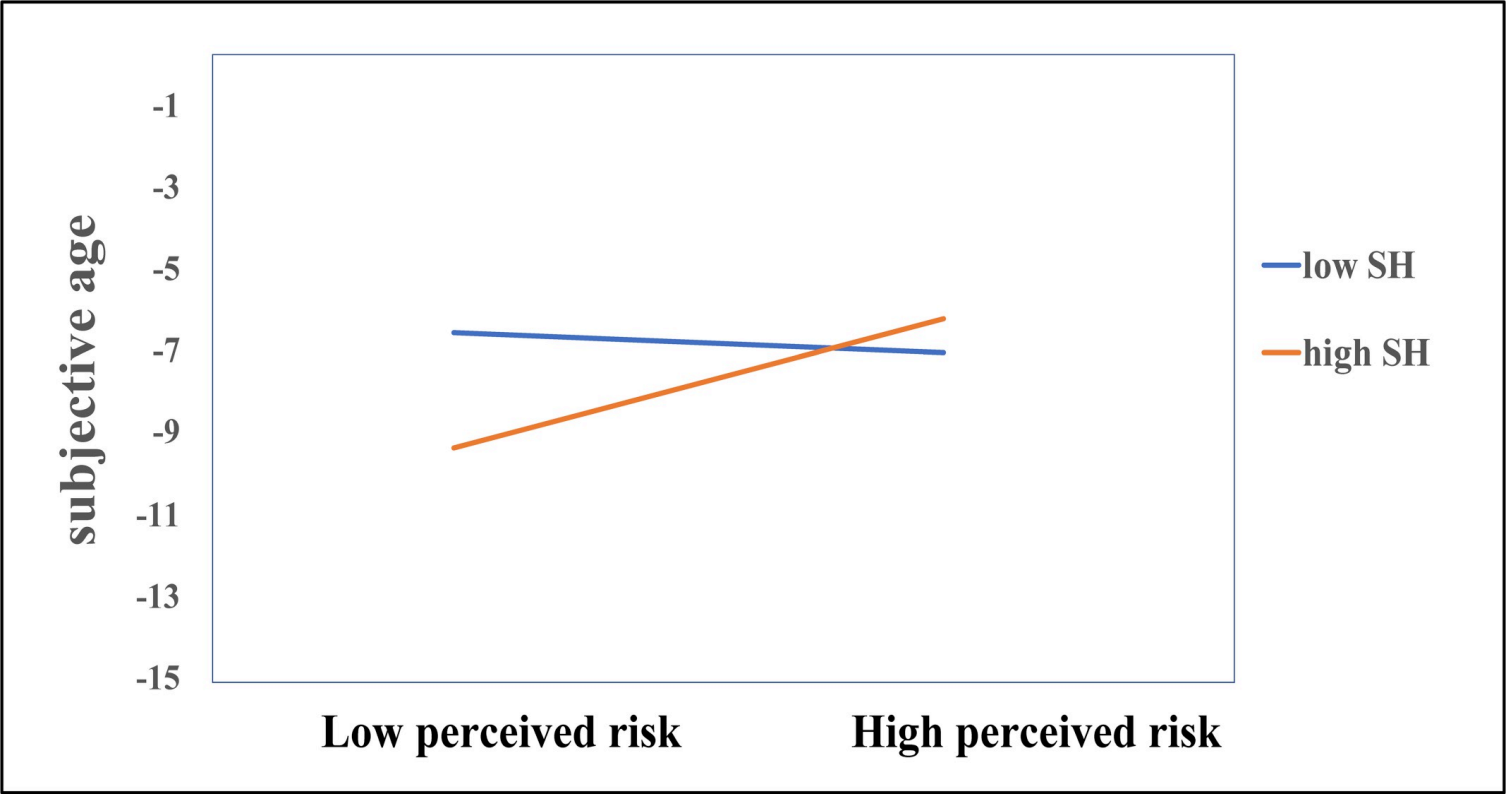
Simple slopes for the effect of perceived risk of serious disease course at T1 predicting subjective age at T2, moderated by subjective health (SH). High and low perceived risk of falling seriously ill and subjective health groups represent values 1 SD above and below the mean, respectively. Analyses are controlled for age, gender, education, and subjective health.

The cross-lagged models for subjective age and perceived risk of serious illness with Covid-19 yielded a significant positive relationship from perceived risk at T1 to subjective age at T2 indicating that those who perceive their risk as higher feel older over time (Table 5, Model 1), and this effect also persisted when entering the covariates (Table 5, Model 2). The covariates age, education and gender did not significantly predict any of the dependent variables in the models including subjective age and perceived risk of falling seriously il (see Table 3 in S3 Appendix). Better subjective health at T1 predicted lower perceived risk of serious illness at T2 (β = -.114, *p* = .010). Subjective health was also a significant moderator of the relationship between perceived risk of falling seriously ill and subjective age (Table 5, Model 3). Simple slopes analyses indicated that risk of falling seriously ill predicted subjective age only for those with better subjective health (*b* = 1.567, *p = <* .001), whereas there was no effect of risk for those with low subjective health (*b* = -0.248, *p =* .555) (Fig 3). Participants with a good subjective health felt younger if they perceived their risk of falling seriously ill as low, and older if they perceived their risk as high. There was a significant correlation between the residuals of the T2 variables in all models including subjective age and perceived risk of falling seriously ill (model 1: *rc =* .107, *p =* .030*)*, indicating that as subjective age increased, so did perceived risk of falling seriously ill, irrespective of bidirectional relationships.

**Table 5:** Standardized estimates for the cross-lagged regression models including subjective age, and perceived risk of falling seriously ill with Covid-19.

	Initial Correlation	Stability		Crossed-lagged effect		Moderator effect		Direct effect		Residual Correlation	
CL Model, SA and PriskS	ʳPriskS↔SA1	SA1↔ SA2	PriskS1→PriskS2	PriskS1→SA2	SA1→PriskS2	PriskS1→SA2	SA1→Prisk2	SH→PriskS2	SH→SA2	PriskS2↔SA2	
1 Simple Model	0.148*	.610*	.584*	.104*	0.029					.104*	
2 Simple Model with Covariates	0.146*	.599*	.533*	.091*	0.019					.105*	
3 Model with Subjective Health	0.147*	611*	.533*	.091*	0.021	.136*	0.005	-.114*	-0.067	.107*	

Models with covariates include age, gender, education and subjective health at timepoint 1. Prisk-S1, perceived risk of serious disease course at timepoint 1; SA1 subjective age at timepoint 1; Prisk-S2, perceived risk of serious disease course at timepoint 2; SA2, subjective age at timepoint 2; SH, subjective health.

*p<0.05.

## Discussion

Given the high relevance of belonging to the risk group of older persons during the Covid-19 pandemic, our study aimed at investigating the bidirectional relationship between how old people feel and their worries and perceptions of risk concerning the virus while also including perceived health status as a moderator. We found that overall, subjective age increased over the course of the surveyed stage of the pandemic, indicating that on average, participants felt older in October 2020 compared to June 2020. A higher subjective age at T1 was cross-sectionally related to a higher perceived risk of serious course of disease, but not to worry or risk of infection. As for longitudinal relationships and contrary to our expectations, neither worry nor perceived risk of contracting Covid-19 showed a significant independent relationship with subjective age in either direction. The relationship of worry and subjective age was dependent on subjective health, with an effect in opposite directions depending on whether subjective health was good or bad. For participants with poor subjective health at T1, the relation was negative meaning that those who worried more at T1 felt younger at T2 compared to those who worried less. For those with better subjective health the relation was positive indicating that if people worried more at T1 they felt older at T2 compared to those who worried less. As expected, perceived risk of falling seriously ill was a significant predictor for subjective age three months later, and health status moderated this relationship: amongst participants with a better subjective health, those who perceived their risk of serious disease as lower in June 2020 felt younger in October 2020, whereas for those with a poor subjective health their perceived risk of falling seriously ill had no effect on subjective age. Finally, we found significant residual correlations in both models including perceived risk, indicating correlates changes in the sense that increases in subjective age are associated with higher level of perceived risk of contracting the virus and of falling seriously ill, irrespective of directional relations.

### The effect of worry and risk perception on subjective age and the moderating role of subjective health

Our results revealed that both worry of falling ill and perceived risk of falling seriously ill predicted subjective age three months later. However, the main effect was only significant for perceived risk of falling seriously ill. Regarding worry, we expected our results to be in line with the distancing hypothesis stating that people who experienced more worry and risk would feel younger over time to distance themselves from the risk group. On the contrary and in contrast to the results of Terracciano, Stephan [29], we found that subjective age increased between measurement occasions as the pandemic progressed, and that overall, worry did not predict subjective age over time. Only for participants with lower perceived health did more worry predict a younger subjective age. For participants with better subjective health, no distancing effect was found, but rather the contrary: more worry increased subjective age for those with good subjective health. Although those with worse health and more worry felt younger compared to those with a poorer health and less worry, there was not a big difference in the subjective ages between good and poor health for those with more worry. Thus, the protective effect of good subjective health on subjective age seems to be leveraged when people worry a lot. It might be interesting to consider this in terms of the effects of risk communication: Those older people who are in good health might have the most to lose and thus be especially susceptible to fear-inducing communication in terms of placing them in the risk group, thus their subjective age increases. Future studies should address this possibility and link it to objective risks of infection, such as number of Covid cases, which also seem to be relevant with regard to subjective age [23].

Higher perceived risk of a serious course of disease did also not lead to distancing tendencies as the original models showed that more perceived risk of a serious course of disease predicted a higher subjective age. The effect became non-significant for participants who experienced their health as worse. Those with a better subjective health felt youngest when they perceived their risk as low, also in contrast to any distancing mechanism. In sum, neither the model with worry, nor the model with perceived risk of a serious course of disease gave clear support for the distancing hypothesis.

One factor that might have played a role regarding the differences of our effects compared to Terracciano and colleagues [29], is that our sample consisted mainly of older people (aged 60+), while their sample was aged 18 and older. People over the age of 60 were mainly lumped into one category of at-risk persons during the pandemic, which might have contributed to the fact that chronological age did not play a major role in our results, compared to the age people felt like. Given these differences, it might be worthwhile to investigate the relations between subjective age and worries as well as risk perception in different stages of the lifespan and also at different points in time during the pandemic. Depending on the stage of the pandemic and the current focus of the discourse, the age-dependency of the relations might be highly dynamic.

Despite the lack of directional effects, we found significant positive residual correlations in models including both risk perceptions, which speaks for correlated changes between the variables beyond any directional relations. Other variables, such as sense of control, might thus be needed to be addressed to understand the relationship between perceived risk and subjective age. For instance, a study by Sesker and colleagues [48], found that perceived control declined significantly during the early onset of the pandemic. As a lower sense of control can make older adults feel older on a daily basis [49], and we know that it is related to both subjective age and risk perceptions [37,49] incorporating sense of control into future analysis, we could gain a better understanding of the mechanisms behind subjective age.

Naturally, perceived health should be highly relevant for people’s worries and risk perception during an ongoing health crisis, especially for older people. Our findings support this as health moderated the relationship of subjective age, worry, and risk perception. Relatedly, Ayalon and Cohn-Schwartz [36] found that people with chronic illnesses tend to worry more about Covid-19, confirming the strong connection between health/illness and worries related to the pandemic. Furthermore, our findings suggest that the associations between subjective age, risk perception and worry are contingent on specific contextual factors, such as subjective health. In our view, the results indicate that the trajectories of subjective age during the pandemic show big interindividual differences and depend on specific compositions of resources and stress factors. These resources and constrains could also be related to different domains. Interestingly, Lanciano and colleagues [37] found that perceived risk in the health domain was rather low compared to other risk perceptions, e.g., in the domain of work or institutional economy during the Covid-19 emergency. In future research it will be interesting to better understand which domains and what factors contribute to younger subjective ages and the way in which subjective age, risk perception and health interact over longer periods of time.

### The effect of subjective age on worry and risk perception

In terms of bidirectional effects, we had hypothesized that subjective age would have an effect on worry and risk perceptions, and that his effect would follow the same trajectories as chronological age in the sense that people who felt younger would worry less and perceive their risk of getting infected with Covid-19 as lower [34,40]. However, we did not find that subjective age predicted worry or perceived risks over the course of three months. Our first model including subjective age and worry yielded a small significant effect from subjective age to worry, but this effect did not hold when covariates were added. Overall, the results indicate that ones’ subjective age may not be considered when worrying or estimating ones’ risks of falling ill with Covid-19. However, as our study time was quite limited (3 months), we were not able to map dynamic relations between variables. There might be phases during the pandemic, when subjective age might be more predictive for people’s worries and risk perceptions, that our design precluded to detect. Given that we found significant correlations at T1 between for instance, subjective age and perceived risk of falling seriously ill, there might have been some adaptations that had already taken place at earlier timepoints. In addition, subjective age may vary and be more potent depending on specific situations. As people most likely answered our questions while staying at home, this might have represented a situation of security and control, which tend to lower subjective age [49], and as such people’s perceptions of risk may be less affected.

Overall, factors other than subjective age might be more predictive of worries and risk perceptions, especially for older adults. We should thus consider for instance people’s more general emotional states and anxiety levels, which we could not control for in the current study. Besides, older adults are found to show more emotional resilience than younger people and this seems to have persisted during the current pandemic [50]. In sum, we should investigate whether there is a shift in which variables best predict worries and risk perceptions depending on the different stages of life after 60, and whether age differences in general tendencies of negative affect and anxiety might play a role in this regard.

### Limitations and directions for future research

Our study adds to previous studies of subjective age during the pandemic by exploring a bidirectional model including subjective age, worries- and perceptions of risk related to Covid-19, and by including health status as a moderator. We have contributed to nuancing the idea of subjective age as a coping mechanism specifically and to the complex nature of how people react to the ongoing pandemic more generally. Finally, our longitudinal data collection with a large sample of older adults was conducted at the beginning of the pandemic outbreak and has in such captured some of the early onset of reactions of older people related to the pandemic.

Some limitations still need to be pointed out which at the same time open directions for future research. As already mentioned, although we use longitudinal data, our data collection is limited to two time points between the first two waves of the covid outbreak in Luxembourg. Given the highly volatile nature of the pandemic, and the development of vaccines that were first offered to older and at-risk individuals, it would have been interesting to see how the relationship between subjective age, worry, and risk perception develops over longer periods of time. Furthermore, our first data collection took place at the end of the first wave, when some adaptations and adjustments might already have taken place. Evidence for the time dependency of effects during the pandemic come from several studies. For instance, Li, Luan [51] found that in a US sample risk perceptions did impact preventive behaviour at the start of the pandemic, but over the course of the pandemic, preventive behaviour increased people’s perception of infection-risk which again contributed to an increase in preventive behaviour. In addition, Niepel, Kranz [39] found that respondents underestimated their fatality risk related to Covid-19 compared to epidemiological figures. However, the estimation became more accurate over time. These findings indicate that the relationships between worry, risk perceptions and other variables can change in strength and direction depending on specific conditions at a given time. Thus, future studies should aim at analysing dynamic, time-lagged, and bidirectional effects across all the waves of the pandemic, numbers of infection [23] and the ongoing governmental restrictions and media images of older people. There is the possibility that the effects of worries, subjective age and health changes or increases over time as the pandemic situation develops, and feedback loops between variables might be possible.

In addition, our study is limited to the situation in Luxembourg. Subjective age has been found to have a stronger effect on health and survival in countries providing less welfare [17]. As our study is conducted in a state providing a high level of welfare than for example, the US, comparing our results to similar studies based on data from disparate countries could provide a better insight into the effect of culture- and environmental factors.

Furthermore, all constructs relevant in the current study were assessed using single items, which might limit the reliability of our results. Subjective age and subjective health are commonly assessed with single items, and those have proven to be quite powerful predictors of e.g., mortality and physical functioning [52,53]. However, even though subjective health is a relevant indicator in concertation with perceived risk, worry and subjective age, additional objective health information might have strengthened our results, but was not at our disposal. Besides, subjective age is a multidimensional construct [21], and thus, future studies should address in how far different dimensions of subjective age, such as ideal age and look age might show differential trajectories and relations.

Finally, in terms of our sample, our study included only participants who were willing and able to answer questions online or via phone. Thus, generalizations to the general or other populations should be considered cautiously.

### Conclusion

Regardless of these limitations, our study is adding to the growing field of research investigating subjective age during the ongoing pandemic. We have shed light on the relationships between subjective age, worry and perceived risks and the role of people’s perceived health. Our study reconfirms that subjective age is a complex construct that can play several roles in the developmental trajectory of aging. Rather rationally, older people are more concerned about having a serious course of disease if sick with Covid-19, than they are about contracting the virus. One’s health status plays a crucial role in how subjective age relates to one’s emotional and cognitive reactions to the pandemic as it can change the direction of effects.

## Supporting information

S1 Appendix Table 1. Results from the cross-lagged model with subjective age, worry, subjective health as a moderator and all covariates. The conceptual model is presented in Fig 1.
(DOCX)

S2 Appendix Table 2. Results from the cross-lagged model with subjective age, perceived risk of infection and subjective health as a moderator and all covariates. The conceptual model is presented in Fig 1.
(DOCX)

S3 Appendix Table 3. Results from the cross-lagged model with subjective age, perceived risk of serious course of disease, subjective health as a moderator and all covariates. The conceptual model in Fig 1.
(DOCX)

S1 Data. Archive (ZIP)
