# Supplementary material for: Subjective age, worry and risk-related perceptions in older adults in times of a pandemic
Source: PLoS One. 2022 Sep 29;17(9):e0274293. doi: 10.1371/journal.pone.0274293 (PMC9522013; doi:10.1371/journal.pone.0274293)
Supplement: S1 Appendix — Results from the cross-lagged model with subjective age, worry, subjective health as a moderator and all covariates. The conceptual model is presented in Fig 1. (DOCX) [file pone.0274293.s001.docx]

**Appendix**

Table 1

*Results from the cross-lagged model with subjective age, worry, subjective health as a moderator and all covariates. The conceptual model is presented in Fig 1.*

T1 Correlations

T1 correlations SA1

Worry1 .111 .009

Age -.116 .006

Edu .036 .427

Gender .014 .738

SH -.290 <.001

T1 correlations Worry1

Age -.054 .180

Edu -.068 .109 Gender -.004 .930

SH -.178 <.001

Cross-lagged results       *β*         SE *β*          *p*

Dependent variable: SA2

SA1 .618 .032 <.000

Worry1 .018 .037 .624

Age .005 .038 .896

Edu .054 .040 .179

Gender -.051 .036 .150

SH -.086 .041 .036

Worry1*SH .137 .040   .001

Dependent variable: Worry2

Worry1 .512 .034 <.001

SA1 .071 .044 .107

Age -.012 .039 .758

Edu .057 .044 .193

Gender -.026 .038 .499

SH -.058 .044 .180

SA1*SH .034 .051 .507

Residual correlation

SA2 with Worry 2 .030 .521

Note. *β* = standardized regression coefficient, SE *β* = standard error *β*, *p* = significance level. SA1, subjective age timepoint 1; SA2, subjective age timepoint 2; Worry1, worry of falling ill with Covid-19 timepoint 1; Worry2, worry of falling ill with Covid-19 timepoint 2; SH, subjective health timepoint 1; Age, chronological age timepoint 1; Edu, education timepoint 1; Gender, participant gender timepoint 1. All control variables at T1 were also correlated with one another.
