# Supplementary material for: Subjective age, worry and risk-related perceptions in older adults in times of a pandemic
Source: PLoS One. 2022 Sep 29;17(9):e0274293. doi: 10.1371/journal.pone.0274293 (PMC9522013; doi:10.1371/journal.pone.0274293)
Supplement: S2 Appendix — Results from the cross-lagged model with subjective age, perceived risk of infection and subjective health as a moderator and all covariates. The conceptual model is presented in Fig 1. (DOCX) [file pone.0274293.s002.docx]

Table 2

*Results from the cross-lagged model with subjective age, perceived risk of infection and subjective health as a moderator and all covariates. The conceptual model is presented in Fig 1.*

T1 Correlations *r p*

T1 correlations SA1

Prisk1 -.012 .794

Age -.115 .006

Edu .037 .404

Gender .010 .810

SH -.286 <.001

T1 correlations Risk1

Age -.042 .312

Edu -.020 .649 Gender .048 .250

SH -.073 .082

Cross-lagged results       *β*         SE *β*          *p*

Dependent variable: SA2

SA1 .604 .040 <.000

Prisk1 -.005 .038 .895

Age .007 .038 .854

Edu .053 .040 .192

Gender .188

SH -.079 .041 .051

Prisk1*SH .062 .043   .149

Dependent variable: Prisk2

Prisk1 .489 .040 <.001

SA1 .000 .048 .993

Age .007 .041 .869

Edu .037 .046 .416

Gender -.006 .041 .880

SH -.013 .046 .780

SA1*SH .021 .053 .695

Residual correlation

SA2 with Prisk2 .154 .001

Note. *β* = standardized regression coefficient, SE *β* = standard error *β*, *p* = significance level. SA1, subjective age timepoint 1; SA2, subjective age timepoint 2; Prisk1, perceived risk of contracting the Covid-19 timepoint 1; Prisk2, perceived risk of contracting Covid-19 timepoint 2; SH, subjective health timepoint 1; Age, chronological age timepoint 1; Edu, education timepoint 1; Gender, participant gender timepoint 1. All Control variables at T1 were also correlated with one another.
