# Supplementary material for: Subjective age, worry and risk-related perceptions in older adults in times of a pandemic
Source: PLoS One. 2022 Sep 29;17(9):e0274293. doi: 10.1371/journal.pone.0274293 (PMC9522013; doi:10.1371/journal.pone.0274293)
Supplement: S3 Appendix — Results from the cross-lagged model with subjective age, perceived risk of serious course of disease, subjective health as a moderator and all covariates. The conceptual model in Fig 1. (DOCX) [file pone.0274293.s003.docx]

Table 3

*Results from the cross-lagged model with subjective age, perceived risk of serious course of disease, subjective health as a moderator and all covariates. The conceptual model is presented in Fig 1.*

T1 Correlations

T1 correlations SA1

Prisk1 -.147 .001

Age -.115 .006

Edu .034 .445

Gender .012 .785

SH -.287 <.001

T1 correlations RiskS1

Age -.150 <.000

Edu -.065 .142 Gender -.046 .277

SH -.368 <.001

Cross-lagged results       *β*         SE *β*          *p*

Dependent variable: SA2

SA1 .611 .032 <.000

PriskS1 .091 .041 .028

Age -.014 .038 .712

Edu .053 .040 .189

Gender -.045 .036 .211

SH -.067 .043 .119

PriskS1*SH .136 .044   .002

Dependent variable: PriskS2

PriskS1 .533 .037 <.001

SA1 .021 .044 .640

Age .067 .040 .089

Edu -.034 .042 .418

Gender .002 .038 .950

SH -.013 .046 .780

SA1*SH .021 .053 .695

Residual correlation

SA2 with PriskS2 .107 .031

Note. *β* = standardized regression coefficient, SE *β* = standard error *β*, *p* = significance level. SA1, Subjective age timepoint 1; SA2, subjective age timepoint 2; PriskS1, perceived risk of contracting the Covid-19 timepoint 1; PriskS2, perceived risk of contracting Covid-19 timepoint 2; SH, subjective health timepoint 1; Age, chronological age timepoint 1; Edu, education timepoint 1; Gender, participant gender timepoint 1.
